# The Egg Cure

**Published:** 1870-07

**Authors:** 


### THE EGG CURE.

It is constantly coming to the notice of the observant that common articles of food are curative of various ailments of man — and beast too.

The white of an egg applied to a burn, not deep, gives immediate relief, because it keeps out the air. Dipping the burnt part in warm or cold water, or covering it with flour, are all efficacious, but the burn may be so situated that it cannot be kept in water; then use the white of the egg.

### EGG OIL.

Boil the egg hard, take out the yolk, crush it, put it over a fire, and keep stirring until it is almost ready to take fire itself, when the oil separates, — two teaspoonfuls from a single yolk. Excellent for curing bruises, scratches and sores. In the book on "Health by Good Living" there are some twenty articles of food mentioned as being curative of various common complaints. See chapter on

"THE FOOD CURE."

